# Understanding the effects of predictability, duration, and spatial pattern of drying on benthic invertebrate assemblages in two contrasting intermittent streams

**DOI:** 10.1371/journal.pone.0193933

**Published:** 2018-03-28

**Authors:** María Mar Sánchez-Montoya, Daniel von Schiller, Gonzalo G. Barberá, Angela M. Díaz, Maria Isabel Arce, Rubén del Campo, Klement Tockner

**Affiliations:** 1 Leibniz-Institute of Freshwater Ecology and Inland Fisheries (IGB), Berlin, Germany; 2 Department of Ecology and Hydrology, Regional Campus of International Excellence “Campus Mare Nostrum”-University of Murcia, Campus de Espinardo, Murcia, Spain; 3 Department of Plant Biology and Ecology, Faculty of Science and Technology, University of the Basque Country (UPV/EHU), Bilbao, Spain; 4 Department of Soil and Water Conservation, CSIC-CEBAS, Campus Universitario de Espinardo. Murcia, Spain; 5 Institute of Biology, Freie Universität, Berlin, Germany; University of Arkansas Fayetteville, UNITED STATES

## Abstract

In the present study, we examined the effects of different drying conditions on the composition, structure and function of benthic invertebrate assemblages. We approached this objective by comparing invertebrate assemblages in perennial and intermittent sites along two intermittent Mediterranean streams with contrasting predictability, duration, and spatial patterns of drying: Fuirosos (high predictability, short duration, downstream drying pattern) and Rogativa (low predictability, long duration, patchy drying pattern). Specifically, we quantified the contribution of individual taxa to those differences, the degree of nestedness, and shifts in the composition, structure and function of benthic invertebrate assemblages along flow intermittence gradients. We observed greater effects of drying on the benthic invertebrate composition in Fuirosos than in Rogativa, resulting in a higher dissimilarity of assemblages between perennial and intermittent sites, as well as a lower degree of nestedness. Furthermore, a higher number of biotic metrics related to richness, abundance and biological traits were significantly different between perennial and intermittent sites in Fuirosos, despite a shorter dry period compared to Rogativa. At the same time, slightly different responses were detected during post-drying (autumn) than pre-drying (spring) conditions in this stream. In Rogativa, shifts in benthic invertebrate assemblages along increasing gradients of flow intermittence were found for three metrics (Ephemeroptera, Plecoptera and Trichoptera (EPT) and Odonata, Coleoptera and Heteroptera (OCH) abundances and aerial active dispersal. Furthermore, we demonstrated that combined gradients of dry period duration and distance to nearest perennial reach can generate complex, and different, responses of benthic invertebrate assemblages, depending on the flow intermittence metric. Our study advances the notion that special attention should be paid to the predictability, duration and spatial patterns of drying in intermittent streams in order to disentangle the effects of drying on benthic invertebrate assemblages, in particular in areas subject to high spatial heterogeneity and temporal variability in drying conditions.

## Introduction

Intermittent streams that periodically cease to flow and dry out are a global phenomenon [1–3]. Recent estimates suggest that more than 50% of the global fluvial network experiences drying [4, 5]. Intermittent watercourses are predominant in the Mediterranean area [6], where streams exhibit a distinct seasonal and inter-annual hydrological variability, with recurrent flood and drought events [7, 8]. Moreover, flow intermittence is expected to increase worldwide due to global warming, land-cover changes, and increased water abstraction for human use [9–11]. Research in intermittent streams provides valuable information to understand and predict ecosystem responses to these environmental changes [12, 13].

Many studies have recognized that drying, defined as the complete disappearance of surface water, can shape instream biological assemblages including microbes [14–16], algae [17–19], macrophytes [20, 21], fishes [22–24] and aquatic invertebrates [25–29], as well as terrestrial invertebrates colonizing the dry stream bed [30, 31]. The dry period has been shown to exert a strong selective pressure controlling the structure, composition and biological traits of benthic invertebrates [8, 26, 32–34]. Consequently, natural drying is a fundamental hydrological determinant of biodiversity *sensu lato* [35]. In concordance, intermittent streams harbor different and often impoverished aquatic invertebrate assemblages, compared to perennial streams [26, 36–40]. Furthermore, invertebrate assemblages in intermittent streams are characterized by biological traits, which increase their resistance against drying (e.g. eggs, coccons, diapauses) and their resilience after dry events (e.g. aerial dispersal mode) [26, 40–42].

Benthic invertebrate assemblages can be affected by the preceding dry period [28, 43–45]. As a result, benthic assemblages are often less diverse and abundant in intermittent than in perennial reaches (but see [42]), forming a nested subset due to the progressive loss of taxa sensitive to drying [28] (but see [44]). Moreover, drying effects could be relaxed with time as flowing conditions progress [28].

Studies along gradients of flow intermittence have emphasized that dry event duration leads to a decrease in benthic invertebrate abundance and richness [28, 35, 43–46], reflecting that duration of drying, as a proxy of dryness severity, plays a key role shaping these assemblages [47]. Similarly, the spatial drying pattern, defined as the distance to perennial reaches, is a strong modulator because perennial sections act as refugia for taxa sensitive to drying, which facilitates post-drying recovery [47]. The effects of dry period duration and distance to perennial reaches have been exclusively analyzed in streams where the duration of dry events increases continuously and concomitantly with distance to perennial reaches. However, it prevents the discrimination of the relative importance of duration and distance [28]. Finally, predictability of dry event timing may lead to an increase in benthic invertebrate richness in intermittent streams [35].

Duration and predictability of drying vary among intermittent streams [47, 48]. For example, in temperate areas the duration of the dry period is generally short, highly predictable, and mostly restricted to summer. However, in arid and semi-arid areas the dry period is often longer, less predictable and frequently occurs beyond the summer. Unpredictable droughts may hamper the ability of organisms to evolve adaptations such as resistance and resilience forms, whereas seasonally predictable droughts could confer stability to assemblages [32]. At the same time, spatial drying patterns can vary substantially among streams. Along some streams, intermittent sections are constrained to the upper, lower or middle sections [32, 49], whereas other streams exhibit a patchier configuration of alternating perennial and intermittent reaches [32]. Different spatial patterns are expected to affect benthic invertebrate assemblages due to the variation in quantity and quality of food resources between perennial and intermittent reaches and the dispersal constrains of benthic invertebrates [28]. However, only scant information on the effects of the spatial configuration is currently available [50].

In the present study, we examined how the predictability, duration and spatial pattern of drying determine the composition, structure and function of benthic invertebrate assemblages. With this purpose, we compared invertebrate assemblages during base flow conditions in two intermittent Mediterranean streams with contrasting drying conditions. First, we analyzed the differences in benthic invertebrate assemblages between perennial and intermittent sites within each stream during the wet phase (spring and autumn) and determined the contribution of individual taxa to these differences. Second, we quantified the degree of nestedness of benthic assemblages within each stream. Third, we studied the relationship between benthic invertebrates and gradients of both dry period duration and distance to nearest perennial reach along intermittent sites. We tested the following four predictions: i) Differences between perennial and intermittent invertebrate assemblages will be more pronounced in the stream with more predictable drying and higher stability; ii) The degree of nestedness will be lower in the stream undergoing higher predictability, given the expected less similar assemblages between intermittent and perennial reaches; iii) Drying effects will be stronger in autumn than in spring, reflecting a relaxing effect of drying with increasing time of flowing conditions; and iv) In streams where the two flow intermittence metrics (i.e. dry period duration and distance to nearest perennial reach) are not correlated, their combined gradients will generate complex responses of benthic invertebrate assemblages.

## Material and methods

### Study area

**Fig 1:**
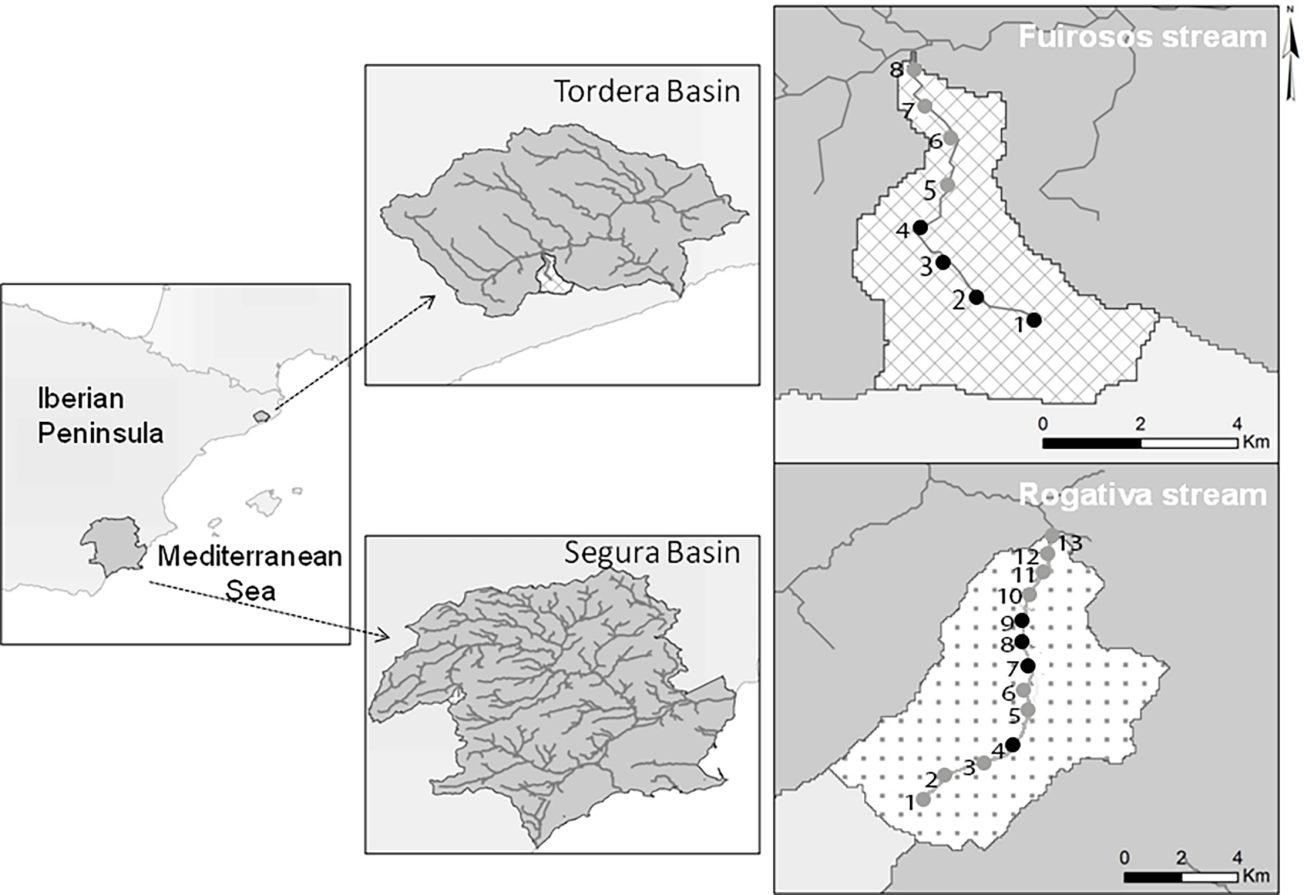
Location and map of the two catchments and the position of individual sampling sites along the two study streams (black dots: Perennial sites; grey dots: Intermittent sites).

The Rogativa (length: 16 km; catchment area: 47.2 km^2^) and Fuirosos (8 km; 15.2 km^2^) are Mediterranean headwater streams located in the Segura and Tordera catchments (Iberian Peninsula; Fig 1). According to the Köppen-Geiger system [51], Rogativa catchment exhibits an arid climate type (BSk; mean annual temperature: 13.3°C, mean annual rainfall: 583 mm) and Fuirosos a temperate type (Csb; 14.3°C, 750 mm).

Both catchments are subject to low human pressure, with more than 90% of the area covered by natural vegetation [31]. In Rogativa, the riparian vegetation is sparse and dominated by helophytes, while the uplands are covered by pines and holm oaks. In Furiosos, the riparian vegetation is dense and dominated by alder and plane trees, while the uplands consist mainly of pine and cork oak forests (see [31] for detailed descriptions).

In both streams flow is temporally and spatially intermittent. Scarce and irregular rain events in Rogativa result in severe and unpredictable dry periods, with some reaches remaining dry for over a year [52]. Fuirosos, however, is subject to a milder and more predictable seasonal drought [53] that ends with the onset of the first major rain events at the end of the summer season, reconnecting the entire river course.

#### Rainfall conditions, flow intermittence characterization and sampling sites

This study was performed between June 2013 and February 2014. Based on the accumulated rainfall over a 12-months period, the year prior to sampling (2012) was very dry in both catchments compared to the previous 5-year period (data from siam.imida.es in Rogativa and www.meteocat.cat in Fuirosos, using climatic stations within 20 km of the basins; S1 Fig). During spring sampling (June 2013), accumulated rainfall was low and similar in both catchments. During post-drying sampling (Rogativa: February 2014; Fuirosos: December 2013), accumulated rainfall was particularly low in Rogativa (S1 Fig). The dry period duration was estimated for each stream using different approaches according to hydrological complexity. In Rogativa, characterized by low predictability, the length of the entire stream was walked at monthly intervals from February 2010 to February 2014 to detect flow cessation and resumption (48 hydrological values for each sampling site). In Fuirosos, with higher predictability, we used temperature sensors to detect flow cessation and resumption [54]. At each sampling site, sensors (iBTag; Alpha Mac Inc.) were deployed in the channel and the riparian zone to continuously record temperature during the entire study period. In both streams, perennial sites were defined as those with surface flow during the entire study period. In addition, in Rogativa, sites exhibiting less than 15% of flow intermittence during the previous 4-years observation period were considered as perennial. The dry period duration was calculated as the number of months (Rogativa) and days (Fuirosos) without surface water during the recording period. The network distance (i.e. the distance along the riverine dendritic network) to nearest perennial reach was calculated for each intermittent site.

Longitudinal spatial drying patterns also differed between streams (Fig 1). Rogativa exhibited a patchy spatial configuration of drying, with alternating perennial and intermittent reaches within its stream network. Conversely, Fuirosos showed a downstream drying gradient with perennial reaches located in the upper sections and intermittent reaches located in the lower sections of the stream network. A total of 13 and 8 sites were selected in Rogativa and Fuirosos, respectively (Fig 1). In Rogativa, 4 sites were located in perennial reaches (1 upstream and 3 downstream reaches) and 9 sites were located in intermittent reaches (Fig 1). In Fuirosos, 4 sites were in the perennial upper section, 4 sites in the intermittent lower section.

#### Invertebrate sampling and metrics

Benthic invertebrates were collected at each sampling site during base flow conditions (reconnected flowing sites) in spring (both streams) and autumn (only in Fuirosos). The first sampling took place before the drying event (Spring: 3 June 2013 in Rogativa, 19 June 2013 in Fuirosos). The second sampling was performed approximately 4 weeks after flow resumption (post-drying: 12 February 2014 in Rogativa, and 13 December 2013 in Fuirosos). In Rogativa, most intermittent sites (n = 8) remained dry during post-drying sampling, preventing the collection of data in autumn.

Following an agreed protocol [28], sampling of benthic invertebrates was carried out at two riffle heads per site to mitigate the effects of small-scale habitat variability. At each riffle head, two samples were randomly collected with a Surber sampler (area: 0.09 m^2^, mesh size: 250 μm) and composited into a single sample. Samples were preserved with 96% ethanol in the field. In the laboratory, all macroinvertebrates were counted and identified at genus level, except for Diptera (family level), Oligochaeta and Ostracoda (class level). A total of 68 composite samples of benthic invertebrates were collected (both streams and dates).

Total, EPT (Ephemeroptera, Plecoptera and Trichoptera), OCH (Odonata, Coleoptera and Heteroptera) and D (Diptera) richness and abundance metrics were calculated for each sample. In addition, the relative proportion of biological traits of benthic invertebrates was calculated. Two trait groups were selected: dispersal (4 categories) and resistance forms (4 categories; cells against desiccation trait was absent), using the databases of [55] and [56]. Additionally, we checked the information about Coleoptera in [57] describing the averaged affinity of each genus to each category. For this study, we rescaled the category affinities (varying from 0 to 5, depending on trait) to 0 to 1 for each trait.

#### Environmental variables

At each sampling site, three periphyton samples were collected from selected rocks (Fuirosos) or from sediment corers (5 cm diameter; Rogativa). The surface area of stones was quantified applying the aluminum foil method [58]. Samples were transported on ice to the laboratory and stored frozen until analysis. The slurry obtained from scraped stones or sediments was filtered using glass fiber filters (Whatman GF/F, Kent, UK). Then, chlorophyll *a* was extracted from filters with 90% acetone overnight at 4°C, and quantified spectrophotometrically (Shimadzu UV1700; Shimadzu Corporation, Kyoto) [58]. In addition, water temperature, pH, salinity, conductivity and dissolved oxygen concentration were measured at each sampling site and date using a handheld sensor (Hach, Loveland, CO, USA). Water column depth and width, and water velocity (current meter MiniAir2; Schiltknecht Co., Zurich, Switzerland) were recorded too. Surface-water discharge was estimated as the product of the average water velocity and cross-sectional area at each sampling site.

#### Data analysis

First, for each stream and season separately, we tested for the effect of flow regime (perennial and intermittent) on assemblage composition using non-metric multidimensional scaling (NMDS) and ANOSIM, on Bray-Curtis similarities matrices based on log-transformed abundances. Later, the Pearson correlation coefficients between environmental variables and axes of the NMDS ordination were calculated. Additionally, we determined the contribution of individual taxa to the differences between flow regime types using the one-way SIMPER routine, aiming to identify which taxonomic groups best typified perennial and intermittent sites.

Second, a nestedness analysis was carried out using Monte Carlo randomization of 400 simulated matrices [59] on presence-absence data to examine whether the invertebrate assemblages at the species-poor sites were nested subsets of the assemblages at the richer sites within each stream.

Third, we performed Generalized Linear Mixed Models (GLMMs; by flexibility with error functions) to test for differences in richness and abundance metrics as well as in the relative proportion of traits between flow regime types (each stream and season separately). We set three levels according to dry event duration (%) (perennial: <15%; moderately intermittent: 15–50% and highly intermittent: >50%). In Rogativa, we explored differences setting the flow regime (fixed factor with three levels: perennial, moderately intermittent and highly intermittent) and site (random factor). In Fuirosos, we tested the effects of flow regime (fixed factor with two levels: perennial and moderately intermittent) and site (random factor) for each season separately. The pairwise differences between the three categories of intermittence on the model only for Rogativa were submitted to posthoc Tukey test. Abundance data were log-transformed as they showed right-skewed distributions, except for abundances by trophic groups, which did not show a similar pattern. The Gaussian error function was assumed for all the variables except for richness ones, as they are counts and thereby we assumed a Poisson error.

Finally, we also used GLMMs to investigate the relationships between richness and abundance metrics and biological traits. and flow intermittence gradients (dry event duration and distance to perennial reach) only in Rogativa, given the low number of intermittent study sites in Fuirosos (n = 4). We fitted the models with dry duration and distance to perennial reach using the spring dataset and site as a random factor. We introduced quadratic terms and interaction between predictors to detect complex non-linear behaviors. We used backward elimination to select the best model for each stream (see S1 Table for steps for model selection in Rogativa stream). Again, Gaussian error function was assumed for all the variables except for richness ones, which were treated with a Poisson error. A valid model including both distance and dry period should not be represented over the full range of both variables because the actual environmental space defined by distance/dry period in the basin is the convex hull shown (S2 Fig), not the rectangle resulting by combining the full ranges. Therefore, we represented and interpreted these models only into the convex hull of the environmental space.

NMDS, ANOSIM and SIMPER analyses were performed with PRIMER v6 [60]. NestCalc was used for the nestedness analysis. GLMM analyses were fitted with the procedure GLIMMIX of SAS 9.4. [61].

## Results

### Flow intermittence characterization

The duration of the dry period was longer and more variable in Rogativa (24–73% of time) than in Fuirosos sites (25–35%) (Table 1). However, the distance of intermittent sites to nearest perennial ones was similar in both streams (Rogativa: 1050-3290m; Fuirosos: 1100-3750m). Dry duration and distance to perennial reach (S2 Fig) were significantly and positively correlated in Fuirosos (Pearson correlation: r = 0.998; P = 0.002), but not in Rogativa (r = 0.584; P = 0.099).

**Table 1:** Flow regime (Per: Perennial, Int: Intermittent) and flow intermittence (proportion of dry period duration, distance to nearest perennial reach) at all sampling sites along the two study streams.

	Rogativa stream			Fuirosos stream		
Site	Flow regime	Flow intermittence		Flow regime	Flow intermittence	
		Dry period (%)	Distance (m)		Dry period (%)	Distance (m)
**1**	Int	68	2930	Per	0	0
**2**	Int	41	1750	Per	0	0
**3**	Int	63	1050	Per	0	0
**4**	Per	13	0	Per	0	0
**5**	Int	24	1580	Int	25	1100
**6**	Int	49	2360	Int	28	2050
**7**	Per	7	0	Int	32	2900
**8**	Per	7	0	Int	35	3750
**9**	Per	14	0			
**10**	Int	48	1160			
**11**	Int	73	2380			
**12**	Int	68	3000			
**13**	Int	73	3290			

### Differences in benthic assemblages between flow regimes

**Fig 2:**
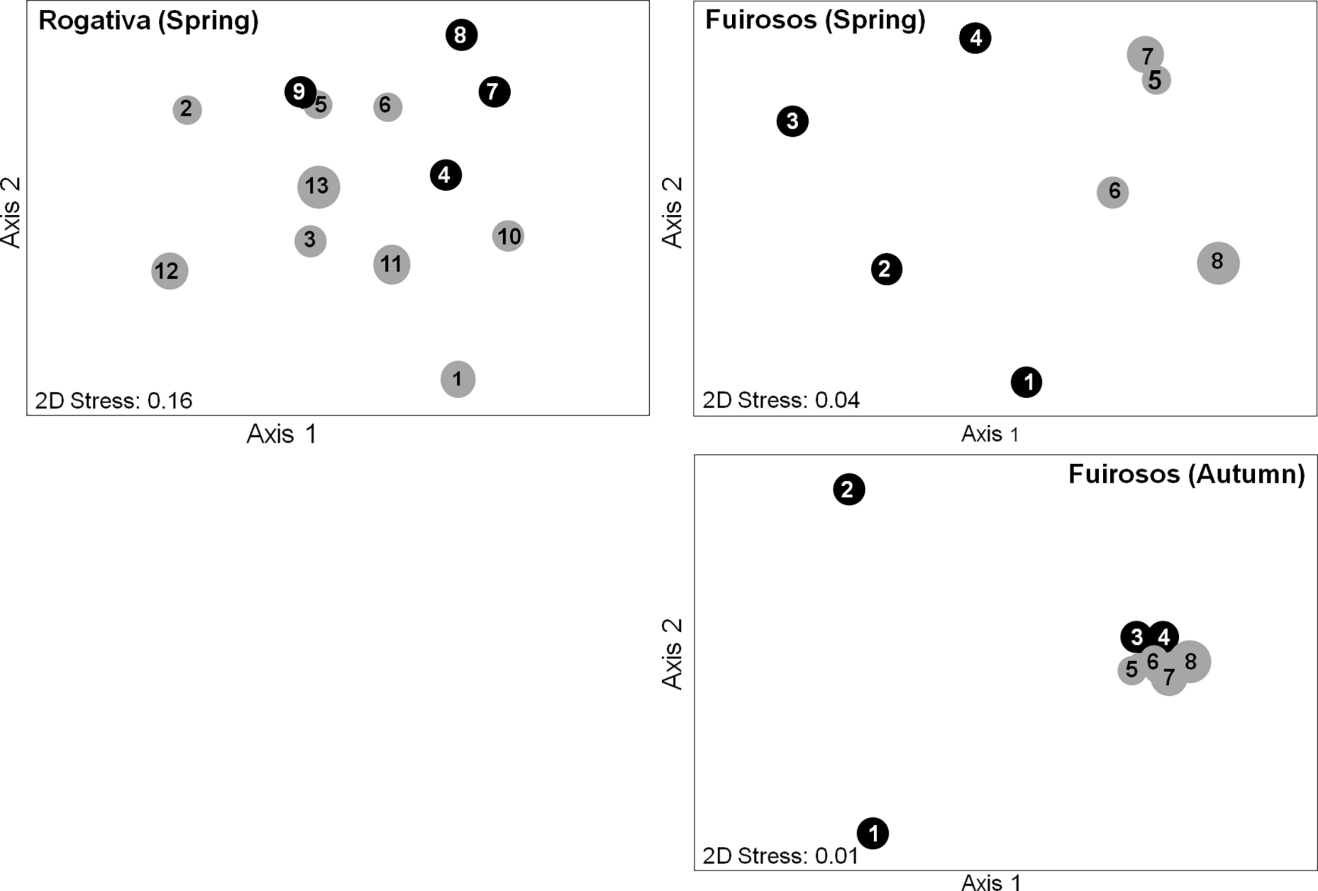
Two-dimensional non-metric multidimensional scaling (NMDS) in the streams Rogativa (spring, left panel) and Fuirosos (spring and autumn, right panels) based on the Bray-Curtis similarities using log-transformed abundance data. Perennial sites: black, intermittent sites: grey. Circle diameter is proportional to flow permanence (Table 1, Fig 1).

A total of 88 taxa was collected in both streams (Rogativa: 52 taxa; Fuirosos: 71 taxa) (S2 Table). In Rogativa, benthic invertebrate assemblages showed a distinct overlap between perennial and intermittent sites in spring (Fig 2), with no significant differences between flow regime types (ANOSIM: R = 0.089; P = 0.29). No correlations were found between environmental variables and the two axes of the NMDS (S3 Table). In Fuirosos, however, invertebrate assemblages were well-separated between perennial and intermittent sites (Fig 2), with significant differences in spring (ANOSIM: R = 0.594; P = 0.029) and in autumn (ANOSIM: R = 0.406; P = 0.029). In spring, conductivity and discharge were significantly and positively correlated to Axis 1 and 2, respectively. In autumn, conductivity was significantly and positively correlated to Axis 1 (S3 Table).

Dissimilarities in assemblage composition were lower in Rogativa than in Fuirosos (both seasons; Table 2). Chironomidae and Simuliidae (Diptera) were the only two common taxa explaining compositional differences in both streams and seasons. In Rogativa, *Baetis* spp. (Ephemeroptera; slightly predominant in perennial sites) also contributed to the dissimilarity between flow regime types (spring). In Fuirosos (spring), *Mercuria* spp. (Gastropoda) and *Sericostoma* spp. (Trichoptera), both predominant in perennial sites, as well as *Oulimnius* spp. (Coleoptera) and *Baetis* spp., both predominant in intermittent sites, contributed to the dissimilarity between flow regime types. Furthermore, in Fuirosos (autumn; with differences slightly higher than in spring) *Capnioneura* spp. (Plecoptera) and *Oulimnius* spp., both predominant in intermittent sites, as well as *Isoperla* spp. (Plecoptera; predominant in perennial sites) explained best the differences.

**Table 2:** Abundance of individual taxa and their contribution to the dissimilarity between flow regime types (perennial and intermittent sites) in Rogativa (spring) and Fuirosos (spring, autumn). Only taxa with a relative contribution of more than 5% to dissimilarity are listed.

Taxa	Average abundance		Contribution to dissimilarity (%)
**Spring (pre-drying)**			
**Rogativa**	Perennial	Intermittent	47.1
Chironomidae	691	2776	41.4
Simuliidae	908	1911	40.6
*Baetis* spp.	248	207	7.6
**Fuirosos**	Perennial	Intermittent	51.9
Chironomidae	1404	2400	33.1
*Oulimnius* spp.	17	582	13.6
*Baetis* spp.	313	747	11.9
*Mercuria* spp.	468	10	11.3
Simuliidae	137	404	7.9
*Sericostoma* spp.	252	0	5.3
**Autumn (post-drying)**			
**Fuirosos**	Perennial	Intermittent	69.1
Simuliidae	757	292	24.8
*Capnioneura* spp.	56	631	24.8
Chironomidae	140	476	15.6
*Oulimnius* spp.	38	342	14.1
*Isoperla* spp.	137	40	5.5

#### Differences in richness, abundance and biological trait metrics between flow regimes

Within each stream, we found significant effects of the flow regime on selected biotic metrics. In Rogativa, only the aerial active trait was significantly different between highly intermittent (lower values) and both perennial and moderately intermittent sites (see S4 Table for values and S5 Table for full tests of fixed effects in Rogativa). In Fuirosos, however, six metrics significantly differed between flow regime types: diapause (higher in perennial sites) and no resistance form (higher in intermittent sites) differed exclusively in spring; EPT richness (higher in perennial sites) and EPT abundance (higher in intermittent sites) differed only in autumn. Finally, OCH abundance and aerial active (higher in intermittent sites) were different in both seasons (see S4 Table for values and S6 Table for full tests of fixed effects in Fuirosos).

### Nestedness in benthic invertebrate assemblages

Invertebrate assemblages were highly nested in both streams and during both seasons, although the degree of nestedness was slightly lower in Fuirosos than in Rogativa (400 permutations, Monte Carlo permutation test, P<0.0001; Rogativa (in spring): T = 21.94, random site-taxon T = 59.5; Fuirosos (in spring) T = 33.78; random site-taxon T = 57.00; Fuirosos (in autumn): T = 34.68, random site-taxon T = 58.13).

### Shifts in benthic invertebrates along flow intermittence gradients (dry period duration and distance to nearest perennial reach)

**Fig 3:**
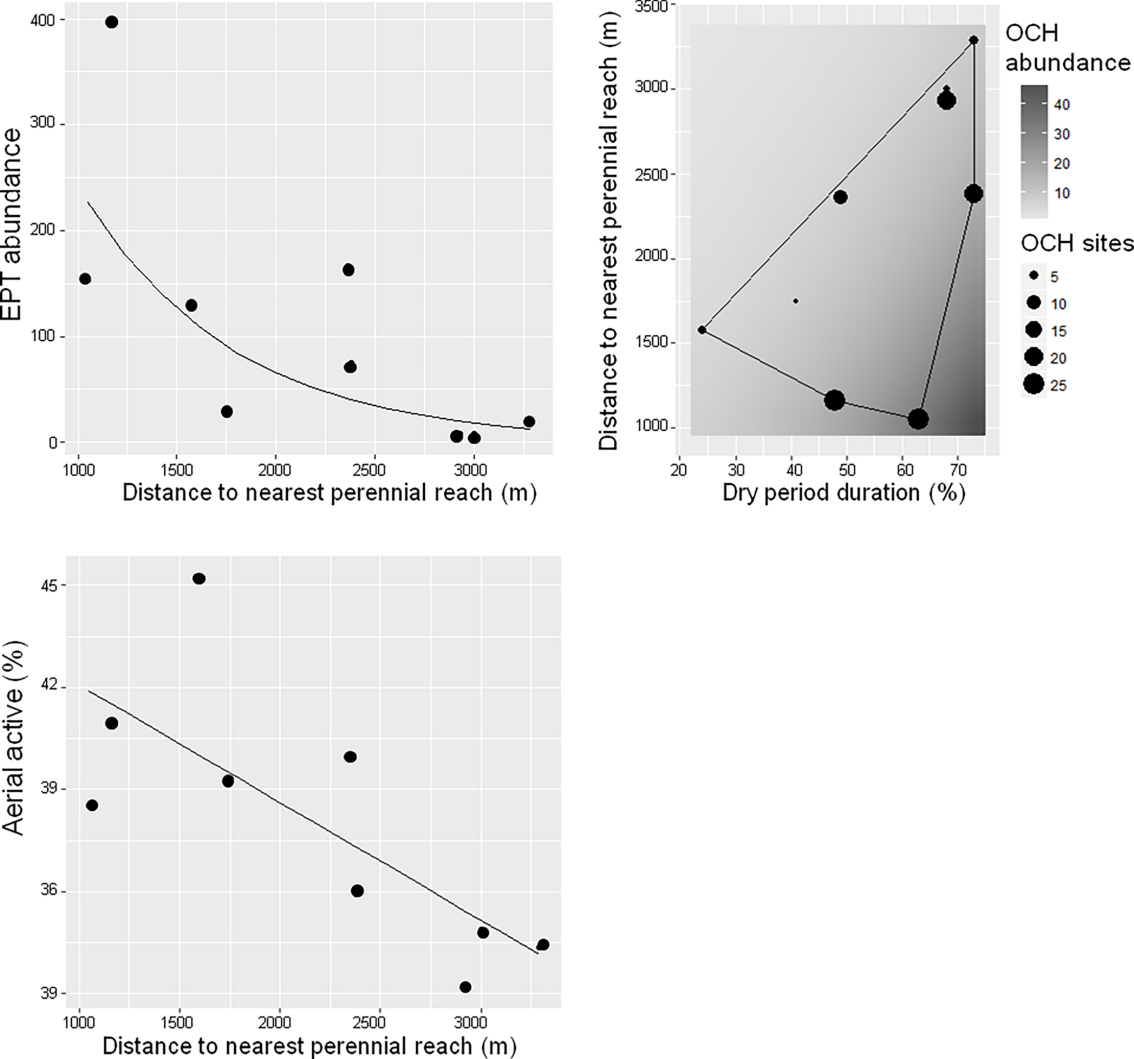
Rogativa: Significant relationship of EPT abundance (upper left panel) and aerial active mode (lower left panel) to distance to nearest perennial reach (m), and of OCH abundance (right panel) to distance and dry period duration (%) in spring. Dots represent empirical data (mean values) per site. On bivariate plots the polygon delimits the convex hull of the actual environmental space. Outside of this polygon, environmental conditions do not exist in the stream and therefore, the model is not applicable.

In Rogativa, three metrics showed a significant relationship with flow intermittence (Fig 3; S7 Table for equations and S8 Table for the full description of the models). EPT abundance and aerial active taxa decreased exponentially and linearly, respectively, with increasing distance to nearest perennial reach. OCH abundance showed a bivariate response, since it increased linearly with increasing dry period duration, but decreased linearly with increasing distance to nearest perennial reach.

## Discussion

During the past few decades, the importance of stream drying for benthic invertebrate assemblages has been increasingly recognized [3]. However, only more recently, the predictability of the timing and duration of dry events have been identified as primary hydrological determinants of biodiversity, similarly to other flow components (e.g. average, low, and high flows) [35]. The present study–by comparing streams of different drying conditions–advances our understanding of the different responses of benthic invertebrate assemblages to drying; emphasizing that predictability, duration, and spatial patterns of drying will require major attention in order to disentangle the effects of drying dynamics on stream biota.

In general, benthic invertebrate richness declines with increasing duration of drying and decreasing predictability of event timing along intermittent streams [35]. This agrees with our comparative analysis that revealed richer assemblages in Fuirosos, with shorter and more predictable dry periods, compared to Rogativa (S4 Table). In addition, Fuirosos exhibited a significantly higher proportion of resistance forms (e.g., eggs, coccons and diapauses) than Rogativa. This is considered a consequence of a higher degree of predictability of drying, which allows benthic invertebrates to establish various adaptations to stream drying [32]. Our findings highlight that the high variability of benthic invertebrate assemblages in intermittent streams during base flow conditions [37] (i.e. presence of hydrological continuity) needs to be considered when comparing benthic invertebrate responses to drying, because those responses may be taxon-specific due to variations in traits of resistance and resilience to drying [32]. In addition, the determination of the degree of variability of benthic invertebrate assemblages remains a major challenge in assessing the ecological status of intermittent streams [37, 62].

Our study revealed significant differences in the composition of benthic invertebrate assemblages between perennial and intermittent sites in Fuirosos, but not in Rogativa. This result supports our prediction that the response to drying would be more pronounced in the stream subjected to more predictable droughts, despite its shorter duration of drying. It must be noticed that different assemblages in Fuirosos were not correlated with algae biomass, eliminating the possibility that food availability could be an important factor explaining those differences [28]. Conversely, homogeneous assemblages in Rogativa along reaches with different flow regimes might be a consequence of the harsh environmental conditions characterised by both a long dry period (up to 70%) and low predictability of drying, which may impose severe filtering in the long term and selection of the taxa most tolerant to drying.

In concordance with the aforementioned results, dissimilarities in benthic invertebrate composition were more pronounced in Fuirosos than in Rogativa. Compositional differences, because of drying, were associated in both streams mainly to two common taxa of dipterans: Chironomids (Chironomidae) and blackflies (Simuliidae) were predominant in intermittent sites, as previously reported [28, 47, 63]. This is likely a consequence of their physiological tolerance to desiccation and of resistance forms to withstand the dry period [64, 65]. In fact, recent studies emphasized that the persistence of Chironomidae in intermittent streams can result from a combination of strategies, including the use of hyporheic zone and moist microhabitats and the development of specific resistance strategies [66]. Moreover, a high abundance of *Baetis* spp. in intermittent sites (only in Fuirosos) is in line with previous observations in a temperate intermittent stream in France [28]. Interestingly, the clear predominance of *Capnioneura* spp. (Plecoptera) in intermittent sites (Fuirosos, autumn) can be attributed to the presence of *Capinoneura mitis* in this stream [67]. This species has a univoltine life cycle with larvae present generally from September to April and absent in spring, which is in concordance with our results, with summer egg diapause as a resistance form, which allows withstanding high temperature and drought [68, 69].

Similarly, structural and functional responses to drying were more pronounced in Fuirosos. In Rogativa, significant differences were only found between the two hydrological extreme types (perennial *vs*. highly intermittent sites) which cover a lengthy drying gradient (73%), while in Fuirosos they occurred at a moderate level of drying (35%). This almost general lack of drying effect in Rogativa once more supports our prediction. It must be noticed that perennial reaches, with surface flow during the entire study period, exhibited a dry period of few months during the four years previous to our study. Hence, taxa with drought adaptation in perennial sites were positively selected by previous drying conditions, resulting in different assemblages only from those which persist after a very long dry event. These findings emphasize the importance of considering not only short-term hydrological conditions, but the history of stream discharge conditions too, which acts as an environmental filter for community assemblages [70–72]. This can be especially important in streams characterized by a strong hydrological variability, typical for arid and semi-arid regions [73, 74].

Drying effects on abundance and richness metrics were detected only in Fuirosos, and biological trait changes were more distinct in Fuirosos than in Rogativa. In agreement with previous studies [27, 28], EPT richness was higher in Fuirosos (perennial sites) because many EPT taxa lack distinct resistant forms to withstand droughts and are therefore highly sensitive to desiccation [55]. However, the previously described life cycle and adaptation to drought of *Capinoneura mitis* would explain the significant differences in EPT abundances, with higher abundances in intermittent sections in autumn. On the other hand, significantly higher abundances of OCH and taxa with aerial active dispersal mode in intermittent sites in Furiosos could be attributed to the high diversity in dispersal mechanism that allows them to escape from harsh conditions during the dry period, including aerial active mode [75]. This dispersal mechanism allows adult flying insects to escape from flow cessation and drying to sites less dry or with surface flow [75, 76]. This facilitates not only within-network but also overland dispersal, the latter considered the main dispersal route in fragmented stream networks [77].

According to our second prediction, in both streams assemblages in intermittent sites were a nested subset of perennial sites, thus sharing most taxa found (>83%). However, a slightly lower level of nestedness was detected in Fuirosos compared with Rogativa. This result may reflect the higher proportion of resistant taxa with adaptation to stream drying in Fuirosos (S4 Table). Similar nestedness patterns have been reported in other intermittent streams [27, 28], revealing that drying may cause a discriminatory loss of taxa sensitive to drying rather than selection for desiccation-resistant specialists. Yet, as previously highlighted [44, 77], a finer taxonomic resolution of the highly diverse Chironomidae and Simuliiade taxa [78, 79], the most abundant taxa explaining differences between intermittent and perennial sites in our study, could have reduced the reported nestedness patterns.

Contrary to our third prediction on the expected relaxing effect of drying with time after flow recovery, results in Fuirosos showed only slightly higher dissimilarities between perennial and intermittent sites in autumn than in spring, as well as similar drying responses in composition and metrics in both seasons. This result implies that in Fuirosos after continuous flow for over 5 months, benthic invertebrate assemblages in intermittent reaches are still different from those in perennial sites. This sustained pattern over time could reflect the high predictability of drying over years, which could favor the observed segregation of assemblages in reaches with different flow regimes.

According to our last prediction, the combined gradients of the two studied flow intermittence metrics (dry period and distance to nearest perennial reach) in Rogativa, where those metrics were not correlated, showed opposite effects on assemblages depending on the flow intermittence metric. More specifically, the response of OCH abundances increased with increasing dry period duration and decreased with increasing distance to nearest perennial reach (Fig 3). This finding may indicate the preference of OCH taxa for highly intermittent reaches likely because of their high affinity for stagnant pool habitats [37, 72, 73] typically abundant in these sites year-round [74]. However, this preference appeared to be limited to sites located close to perennial reaches likely due to dispersal limitations. As previously reported in other intermittent streams [28, 45, 77], EPT abundances and taxa with aerial active dispersal mode also decreased with distance to nearest perennial reaches, suggesting that dispersal limitation played an important role in shaping assemblage in Rogativa stream.

In conclusion, predictability, duration and spatial patterns of drying must be considered when disentangling the effects of drying on benthic invertebrate assemblages in intermittent streams; especially in areas subjected to a high natural variability of drought conditions. Global change and increasing water use will increase the spatial heterogeneity and temporal variation in predictability and severity of drying events worldwide. Hence, more attention must be paid to the various components of the drying regime in order to achieve a more comprehensive understanding of the implications of flow intermittence on biotic assemblages.

## Supporting information

S1 Fig Accumulated rainfall in the previous 12 months in the two study streams (June 2008-March 2014). Vertical dotted line indicates pre-drying (spring) sampling time (June 2013 in both streams) and vertical dot-dashed line indicates post-drying (autumn) sampling time in Rogativa (February 2014) and Fuirosos (December 2013).
(TIF)

S2 Fig Relationship between dry period duration (%) and distance to nearest perennial reach (m) in the two study streams. Regression lines between both variables and their 95% confidence intervals (lighter grey). The darker grey polygon delimits the studied environmental space on Rogativa as defined by the two variables calculated as the convex hull (see Methods).
(TIF)

S1 Table Strategy for fitting models on intermittent reaches to gradients of dry period duration (Dry) and distance to nearest perennial site (Dis) in each study season in the Rogativa and Fuirosos streams. (DOCX)

S2 Table Abundance of benthic invertebrate taxa in the study sites in Rogativa (spring) and Fuirosos (spring and autumn) streams. (XLSX)

S3 Table Pearson’s correlations between the two NMDS ordination axes and the studied environmental variables in Rogativa and Fuirosos streams. Values in italics indicate statistical significance at P<0.05.
(DOCX)

S4 Table Least square means (± SE) values by stream, season and flow regime as calculated from models for Rogativa and Fuirosos streams independently. (EPT: Ephemeroptera, Plecoptera and Trichoptera, OCH: Odonata, Coleoptera and Heteroptera and D: Diptera)
(DOCX)

S5 Table Results of GLMMs for flow regime effect (P = perennial, M = moderately intermittent, H = highly intermittent) in Rogativa stream in spring. Values in italics indicate statistical significance at P<0.05. (EPT: Ephemeroptera, Plecoptera and Trichoptera, OCH: Odonata, Coleoptera and Heteroptera and D: Diptera).
(DOCX)

S6 Table Results of GLMMs for flow regime effect (perennial, intermittent) in Fuirosos stream in spring and autumn separately. Values in italics indicate statistical significance at P<0.05. (EPT: Ephemeroptera, Plecoptera and Trichoptera, OCH: Odonata, Coleoptera and Heteroptera and D: Diptera).
(DOCX)

S7 Table Equations for models relating distance to nearest perennial reach (Dis; km) and dry period duration (Dry; %) for benthic invertebrate metrics in Rogativa stream. (EPT: Ephemeroptera, Plecoptera and Trichoptera, OCH: Odonata, Coleoptera and Heteroptera).
(DOCX)

S8 Table Mixed models for only significant relationships between dry period duration (dry) and distance to nearest perennial reach (distance) with benthic invertebrate metrics in intermittent sites in Rogativa stream. (EPT: Ephemeroptera, Plecoptera and Trichopetera, OCH: Odonata, Coleoptera and Heteroptera).
(DOCX)
