# Supplementary figures and images for: Understanding the effects of predictability, duration, and spatial pattern of drying on benthic invertebrate assemblages in two contrasting intermittent streams
Source: PLoS One. 2018 Mar 28;13(3):e0193933. doi: 10.1371/journal.pone.0193933 (PMC5874014; doi:10.1371/journal.pone.0193933)

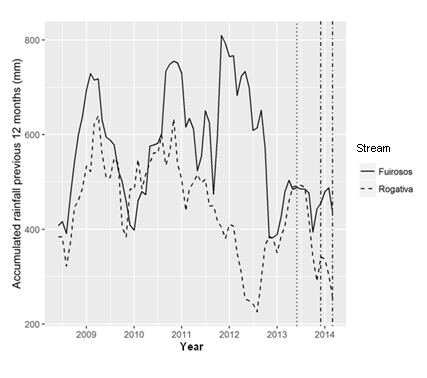

Supplement: S1 Fig — Vertical dotted line indicates pre-drying (spring) sampling time (June 2013 in both streams) and vertical dot-dashed line indicates post-drying (autumn) sampling time in Rogativa (February 2014) and Fuirosos (December 2013). (TIF) [file pone.0193933.s001.tif]

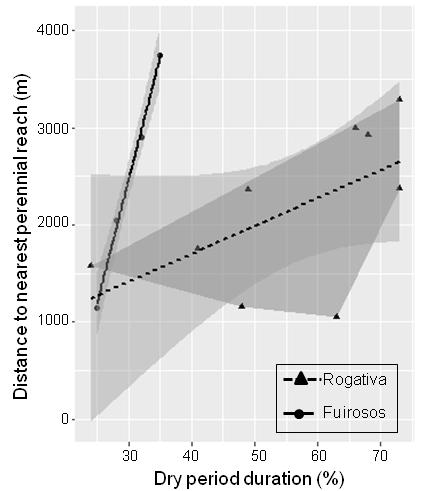

Supplement: S2 Fig — Regression lines between both variables and their 95% confidence intervals (lighter grey). The darker grey polygon delimits the studied environmental space on Rogativa as defined by the two variables calculated as the convex hull (see Methods). (TIF) [file pone.0193933.s002.tif]
