# Supplementary material for: Understanding the effects of predictability, duration, and spatial pattern of drying on benthic invertebrate assemblages in two contrasting intermittent streams
Source: PLoS One. 2018 Mar 28;13(3):e0193933. doi: 10.1371/journal.pone.0193933 (PMC5874014; doi:10.1371/journal.pone.0193933)
Supplement: S1 Table — (DOCX) [file pone.0193933.s003.docx]

**S1 Table.**

|  | **Rogativa** | **Fuirosos** |
| --- | --- | --- |
| ***Step 1*** | Dry + Dis + Dry^2^ + Dis^2^ + Dry×Dis | Dis+Dis^2^ |
| ***Step 2*** | Dry + Dis + Dry^2^ + Dry×Dis *and* Dry + Dis + Dis^2^ + Dry×Dis | Dis |
| ***Step 3*** | Dry + Dis + Dry×Dis |  |
| ***Step 4*** | Dry + Dis |  |
| ***Step 5*** | Dry *and* Dis |  |
