# Supplementary material for: Understanding the effects of predictability, duration, and spatial pattern of drying on benthic invertebrate assemblages in two contrasting intermittent streams
Source: PLoS One. 2018 Mar 28;13(3):e0193933. doi: 10.1371/journal.pone.0193933 (PMC5874014; doi:10.1371/journal.pone.0193933)
Supplement: S3 Table — Values in italics indicate statistical significance at P<0.05. (DOCX) [file pone.0193933.s005.docx]

**S3 Table**.

| **NMDS axis** | **Environmental variable** | **r-values** | **P** | **r-values** | **P** |
| --- | --- | --- | --- | --- | --- |
| **Rogativa (Spring)** |  | **Axis 1** | | **Axis 2** | |
|  | Water temperature | -0.208 | 0.496 | -0.513 | 0.073 |
|  | pH | -0.180 | 0.556 | -0.450 | 0.122 |
|  | Conductivity | 0.338 | 0.259 | 0.246 | 0.418 |
|  | Dissolved oxygen | -0.310 | 0.303 | 0.572 | 0.053 |
|  | Discharge | 0.544 | 0.055 | 0.543 | 0.055 |
|  | Chlorophyll a | 0.119 | 0.700 | 0.498 | 0.083 |
| **Fuirosos (Spring)** | Water temperature | -0.090 | 0.831 | 0.171 | 0.685 |
|  | pH | 0.191 | 0.651 | 0.453 | 0.259 |
|  | Conductivity | 0.759 | *0.029* | 0.090 | 0.831 |
|  | Dissolved oxygen | -0.007 | 0.987 | 0.486 | 0.222 |
|  | Discharge | 0.122 | 0.774 | 0.880 | *0.004* |
|  | Chlorophyll a | -0.457 | 0.255 | -0.060 | 0.888 |
| **Fuirosos (Autumn)** | Water temperature | -0.613 | 0.106 | 0.015 | 0.973 |
|  | pH | -0.116 | 0.784 | -0.037 | 0.931 |
|  | Conductivity | 0.900 | *0.002* | -0.177 | 0.674 |
|  | Dissolved oxygen | 0.676 | 0.066 | -0.286 | 0.492 |
|  | Discharge | 0.264 | 0.527 | 0.042 | 0.921 |
|  | Chlorophyll a | 0.539 | 0.168 | 0.070 | 0.868 |
