# Supplementary material for: Understanding the effects of predictability, duration, and spatial pattern of drying on benthic invertebrate assemblages in two contrasting intermittent streams
Source: PLoS One. 2018 Mar 28;13(3):e0193933. doi: 10.1371/journal.pone.0193933 (PMC5874014; doi:10.1371/journal.pone.0193933)
Supplement: S4 Table — (EPT: Ephemeroptera, Plecoptera and Trichoptera, OCH: Odonata, Coleoptera and Heteroptera and D: Diptera) (DOCX) [file pone.0193933.s006.docx]

**S4 Table.**

|  | **Rogativa** | | | **Fuirosos** | | | |
| --- | --- | --- | --- | --- | --- | --- | --- |
|  | **Spring** | | | **Spring** | | **Autumn** | |
|  | **Perennial** | **Moderately intermittent** | **Highly intermittent** | **Perennial** | **Moderately Intermittent** | **Perennial** | **Moderately Intermittent** |
| **Total richness (log)** | 2.59 ± 0.12 | 2.58 ± 0.11 | 2.44 ± 0.11 | 2.85 ± 0.10 | 2.74 ± 0.11 | 2.89 ± 0.10 | 2.60 ± 0.11 |
| **EPT richness (log)** | 1.42 ± 0.19 | 1.06 ± 0.21 | 0.80 ± 0.23 | 1.80 ± 0.18 | 1.72 ± 0.13 | 1.88 ± 0.17 | 1.32 ± 0.21 |
| **OCH richness (log)** | 1.00 ± 0.23 | 1.25 ± 0.19 | 1.14 ± 0.19 | 1.18 ± 0.20 | 1.23 ± 0.21 | 1.01 ± 0.22 | 1.01 ± 0.22 |
| **D richness (log)** | 1.49 ± 0.18 | 1.61 ± 0.16 | 1.44 ± 0.16 | 1.53 ± 0.16 | 1.49 ± 0.18 | 1.58 ± 0.16 | 1.39 ± 0.18 |
| **Total abundance (log)** | 6.78 ± 0.38 | 7.15 ± 0.37 | 7.70 ± 0.34 | 7.16 ± 0.30 | 7.54 ± 0.31 | 6.26 ± 0.30 | 6.53 ± 0.30 |
| **EPT abundance (log)** | 4.82 ± 0.53 | 4.47 ± 0.53 | 3.34 ± 0.47 | 5.46 ± 0.29 | 5.91 ± 0.31 | 4.64 ± 0.29 | 5.68 ± 0.29 |
| **OCH abundance (log)** | 2.07 ± 0.34 | 2.17 ± 0.33 | 2.54 ± 0.30 | 2.68 ± 0.55 | 5.15 ± 0.57 | 2.17 ± 0.55 | 4.59 ± 0.55 |
| **D abundance (log)** | 6.56 ± 0.39 | 7.01 ± 0.38 | 7.66 ± 0.35 | 6.52 ± 0.40 | 6.97 ± 0.42 | 5.60 ± 0.40 | 5.40 ± 0.40 |
| **Aquatic passive** | 69.23 ± 1.31 | 67.76 ± 1.27 | 66.59 ± 1.16 | 64.63 ± 1.20 | 64.05 ± 1.28 | 70.87 ± 1.20 | 70.52 ± 1.20 |
| **Aquatic active** | 53.22 ± 3.79 | 49.13 ± 3.97 | 49.49 ± 3.37 | 56.25 ± 1.97 | 53.07 ± 2.10 | 39.91 ± 1.97 | 43.70 ± 1.97 |
| **Aerial passive** | 73.58 ± 4.12 | 71.97 ± 4.05 | 80.22 ± 3.67 | 56.58 ± 5.67 | 27.12 ± 6.07 | 47.39 ± 5.67 | 46.43 ± 5.67 |
| **Aerial active** | 42.99 ± 1.97 | 41.35 ± 1.90 | 35.41 ± 1.75 | 39.09 ± 3.51 | 52.94 ± 3.64 | 36.89 ± 3.51 | 52.04 ± 3.51 |
| **Eggs and statoblasts** | 25.65 ± 4.13 | 20.11 ± 4.02 | 17.15 ± 3.67 | 31.67 ± 3.10 | 36.43 ± 3.32 | 11.67 ± 3.10 | 19.91 ± 3.10 |
| **Cocoons** | 1.52 ± 0.39 | 0.97± 0.37 | 0.47 ± 0.34 | 3.87 ± 1.53 | 1.43 ± 1.60 | 1.35 ± 1.53 | 1.62 ± 1.53 |
| **Diapause or dormancy** | 16.26 ± 3.50 | 13.19 ± 3.43 | 15.76 ± 3.12 | 26.03 ± 4.37 | 7.06 ± 4.50 | 16.73 ± 4.37 | 3.94 ± 4.37 |
| **No form of resistance** | 45.25 ± 9.77 | 52.99 ± 9.53 | 51.57 ± 8.70 | 27.49 ± 5.71 | 54.19 ± 5.98 | 71.56 ± 5.71 | 78.10 ± 5.71 |
|  |  |  |  |  |  |  |  |
