# Supplementary material for: Understanding the effects of predictability, duration, and spatial pattern of drying on benthic invertebrate assemblages in two contrasting intermittent streams
Source: PLoS One. 2018 Mar 28;13(3):e0193933. doi: 10.1371/journal.pone.0193933 (PMC5874014; doi:10.1371/journal.pone.0193933)
Supplement: S5 Table — Values in italics indicate statistical significance at P<0.05. (EPT: Ephemeroptera, Plecoptera and Trichoptera, OCH: Odonata, Coleoptera and Heteroptera and D: Diptera). (DOCX) [file pone.0193933.s007.docx]

**S5 Table.**

| **Metrics** | **Type III Tests of Fixed Effects** | | | | |  |  |
| --- | --- | --- | --- | --- | --- | --- | --- |
| **Total richness** | **Effect** | **Num DF** | **Den DF** | **F Value** | **Pr > F** |  |  |
|  | Flow regime | 2 | 11 | 0.58 | 0.5737 |  |  |
|  | **Flow regime** | **Flow regime** | **Estimate** | **Standard Error** | **DF** | **t Value** | **Pr > \|t\|** |
|  | H | M | -0.1436 | 0.1554 | 11 | -0.92 | 0.3754 |
|  | H | P | -0.1499 | 0.1600 | 11 | -0.94 | 0.3689 |
|  | M | P | -0.00636 | 0.1609 | 11 | -0.04 | 0.9692 |
| **EPT richness** | **Effect** | **Num DF** | **Den DF** | **F Value** | **Pr > F** |  |  |
|  | Flow regime | 2 | 11 | 2.32 | 0.1446 |  |  |
|  | **Flow regime** | **Flow regime** | **Estimate** | **Standard Error** | **DF** | **t Value** | **Pr > \|t\|** |
|  | H | M | -0.2583 | 0.3104 | 11 | -0.83 | 0.4230 |
|  | H | P | -0.6245 | 0.2957 | 11 | -2.11 | 0.0584 |
|  | M | P | -0.3661 | 0.2849 | 11 | -1.28 | 0.2252 |
| **OCH richness** | **Effect** | **Num DF** | **Den DF** | **F Value** | **Pr > F** |  |  |
|  | Flow regime | 2 | 11 | 0.37 | 0.7005 |  |  |
|  | **Flow regime** | **Flow regime** | **Estimate** | **Standard Error** | **DF** | **t Value** | **Pr > \|t\|** |
|  | H | M | -0.1178 | 0.2673 | 11 | -0.44 | 0.6680 |
|  | H | P | 0.1365 | 0.2972 | 11 | 0.46 | 0.6551 |
|  | M | P | 0.2542 | 0.2972 | 11 | 0.86 | 0.4106 |
| **D richness** | **Effect** | **Num DF** | **Den DF** | **F Value** | **Pr > F** |  |  |
|  | Flow regime | 2 | 11 | 0.30 | 0.7500 |  |  |
|  | **Flow regime** | **Flow regime** | **Estimate** | **Standard Error** | **DF** | **t Value** | **Pr > \|t\|** |
|  | H | M | -0.1691 | 0.2265 | 11 | -0.75 | 0.4711 |
|  | H | P | -0.04772 | 0.2420 | 11 | -0.20 | 0.8473 |
|  | M | P | 0.1214 | 0.2393 | 11 | 0.51 | 0.6220 |
| **Total abundance** | **Effect** | **Num DF** | **Den DF** | **F Value** | **Pr > F** |  |  |
|  | Flow regime | 2 | 11 | 1.68 | 0.2314 |  |  |
|  | **Flow regime** | **Flow regime** | **Estimate** | **Standard Error** | **DF** | **t Value** | **Pr > \|t\|** |
|  | H | M | 0.5456 | 0.5060 | 11 | 1.08 | 0.3040 |
|  | H | P | 0.9229 | 0.5110 | 11 | 1.81 | 0.0983 |
|  | M | P | 0.3774 | 0.5348 | 11 | 0.71 | 0.4951 |
| **EPT abundance** | **Effect** | **Num DF** | **Den DF** | **F Value** | **Pr > F** |  |  |
|  | Flow regime | 2 | 11 | 2.84 | 0.1014 |  |  |
|  | **Flow regime** | **Flow regime** | **Estimate** | **Standard Error** | **DF** | **t Value** | **Pr > \|t\|** |
|  | H | M | -14.016 | 0.7084 | 11 | -1.98 | 0.0734 |
|  | H | P | -14.746 | 0.7122 | 11 | -2.07 | 0.0627 |
|  | M | P | -0.07299 | 0.7478 | 11 | -0.10 | 0.9240 |
| **OCH abundance** | **Effect** | **Num DF** | **Den DF** | **F Value** | **Pr > F** |  |  |
|  | Flow regime | 2 | 11 | 0.64 | 0.5441 |  |  |
|  | **Flow regime** | **Flow regime** | Estimate | Standard Error | DF | t Value | Pr > \|t\| |
|  | H | M | 0.3766 | 0.4489 | 11 | 0.84 | 0.4193 |
|  | H | P | 0.4779 | 0.4518 | 11 | 1.06 | 0.3128 |
|  | M | P | 0.1013 | 0.4740 | 11 | 0.21 | 0.8348 |
| **D abundance** | **Effect** | **Num DF** | **Den DF** | **F Value** | **Pr > F** |  |  |
|  | Flow regime | 2 | 11 | 2.30 | 0.1464 |  |  |
|  | **Flow regime** | **Flow regime** | **Estimate** | **Standard Error** | **DF** | **t Value** | **Pr > \|t\|** |
|  | H | M | 0.6487 | 0.5164 | 11 | 1.26 | 0.2351 |
|  | H | P | 11.042 | 0.5217 | 11 | 2.12 | 0.0579 |
|  | M | P | 0.4556 | 0.5458 | 11 | 0.83 | 0.4216 |
| **Aquatic passive** | **Effect** | **Num DF** | **Den DF** | **F Value** | **Pr > F** |  |  |
|  | Flow regime | 2 | 11 | 1.13 | 0.3572 |  |  |
|  | **Flow regime** | **Flow regime** | **Estimate** | **Standard Error** | **DF** | **t Value** | **Pr > \|t\|** |
|  | H | M | -11.631 | 17.251 | 11 | -0.67 | 0.5141 |
|  | H | P | -26.331 | 17.500 | 11 | -1.50 | 0.1606 |
|  | M | P | -14.700 | 18.255 | 11 | -0.81 | 0.4377 |
| **Aquatic active** | **Effect** | **Num DF** | **Den DF** | **F Value** | **Pr > F** |  |  |
|  | Flow regime | 2 | 11 | 0.37 | 0.6985 |  |  |
|  | **Flow regime** | **Flow regime** | **Estimate** | **Standard Error** | **DF** | **t Value** | **Pr > \|t\|** |
|  | H | M | 0.3548 | 50.060 | 11 | 0.07 | 0.9448 |
|  | H | P | -37.324 | 50.739 | 11 | -0.74 | 0.4774 |
|  | M | P | -40.872 | 52.959 | 11 | -0.77 | 0.4565 |
| **Aerial passive** | **Effect** | **Num DF** | **Den DF** | **F Value** | **Pr > F** |  |  |
|  | Flow regime | 2 | 11 | 1.31 | 0.3077 |  |  |
|  | **Flow regime** | **Flow regime** | **Estimate** | **Standard Error** | **DF** | **t Value** | **Pr > \|t\|** |
|  | H | M | 82.506 | 54.654 | 11 | 1.51 | 0.1593 |
|  | H | P | 66.382 | 55.153 | 11 | 1.20 | 0.2540 |
|  | M | P | -16.123 | 57.749 | 11 | -0.28 | 0.7853 |
| **Aerial active** | **Effect** | **Num DF** | **Den DF** | **F Value** | **Pr > F** |  |  |
|  | Flow regime | 2 | 11 | 4.78 | 0.0321 |  |  |
|  | **Flow regime** | **Flow regime** | **Estimate** | **Standard Error** | **DF** | **t Value** | **Pr > \|t\|** |
|  | H | M | -59.364 | 25.858 | 11 | -2.30 | ***0.0423*** |
|  | H | P | -75.817 | 26.327 | 11 | -2.88 | ***0.0150*** |
|  | M | P | -16.453 | 27.390 | 11 | -0.60 | 0.5602 |
| **Eggs and statobalsts** | **Effect** | **Num DF** | **Den DF** | **F Value** | **Pr > F** |  |  |
|  | Flow regime | 2 | 11 | 1.20 | 0.3389 |  |  |
|  | **Flow regime** | **Flow regime** | **Estimate** | **Standard Error** | **DF** | **t Value** | **Pr > \|t\|** |
|  | H | M | -29.568 | 54.475 | 11 | -0.54 | 0.5981 |
|  | H | P | -85.006 | 55.276 | 11 | -1.54 | 0.1523 |
|  | M | P | -55.438 | 57.648 | 11 | -0.96 | 0.3569 |
| **Coccons** | **Effect** | **Num DF** | **Den DF** | **F Value** | **Pr > F** |  |  |
|  | Flow regime | 2 | 11 | 2.03 | 0.1773 |  |  |
|  | **Flow regime** | **Flow regime** | **Estimate** | **Standard Error** | **DF** | **t Value** | **Pr > \|t\|** |
|  | H | M | -0.5000 | 0.5077 | 11 | -0.98 | 0.3459 |
|  | H | P | -10.564 | 0.5243 | 11 | -2.01 | 0.0690 |
|  | M | P | -0.5564 | 0.5400 | 11 | -1.03 | 0.3250 |
| **Diapause or dormancy** | **Effect** | **Num DF** | **Den DF** | **F Value** | **Pr > F** |  |  |
|  | Flow regime | 2 | 11 | 0.23 | 0.7959 |  |  |
|  | **Flow regime** | **Flow regime** | **Estimate** | **Standard Error** | **DF** | **t Value** | **Pr > \|t\|** |
|  | H | M | 25.736 | 46.373 | 11 | 0.55 | 0.5900 |
|  | H | P | -0.5032 | 46.908 | 11 | -0.11 | 0.9165 |
|  | M | P | -30.768 | 49.032 | 11 | -0.63 | 0.5431 |
| **No form of resistance** | **Effect** | **Num DF** | **Den DF** | **F Value** | **Pr > F** |  |  |
|  | Flow regime | 2 | 11 | 0.18 | 0.8349 |  |  |
|  | **Flow regime** | **Flow regime** | Estimate | Standard Error | DF | t Value | Pr > \|t\| |
|  | H | M | -14.191 | 129.019 | 11 | -0.11 | 0.9144 |
|  | H | P | 63.224 | 130.829 | 11 | 0.48 | 0.6384 |
|  | M | P | 77.415 | 136.509 | 11 | 0.57 | 0.5820 |
