# Supplementary material for: Understanding the effects of predictability, duration, and spatial pattern of drying on benthic invertebrate assemblages in two contrasting intermittent streams
Source: PLoS One. 2018 Mar 28;13(3):e0193933. doi: 10.1371/journal.pone.0193933 (PMC5874014; doi:10.1371/journal.pone.0193933)
Supplement: S6 Table — Values in italics indicate statistical significance at P<0.05. (EPT: Ephemeroptera, Plecoptera and Trichoptera, OCH: Odonata, Coleoptera and Heteroptera and D: Diptera). (DOCX) [file pone.0193933.s008.docx]

**S6 Table.**

|  |  | **Type III Tests of Fixed Effects** | | | | |
| --- | --- | --- | --- | --- | --- | --- |
| **Metric** | **Season** | **Effect** | **Num DF** | **Den DF** | **F value** | **Pr > F** |
| Total richness | Spring | Flow regime | 1 | 7 | 0.53 | 0.4886 |
|  | Autumn | Flow regime | 1 | 8 | 4.32 | 0.0713 |
| EPT richness | Spring | Flow regime | 1 | 7 | 0.17 | 0.6930 |
|  | Autumn | Flow regime | 1 | 8 | 6.20 | ***0.0325*** |
| OCH richness | Spring | Flow regime | 1 | 7 | 0.04 | 0.8555 |
|  | Autumn | Flow regime | 1 | 8 | 0.03 | 0.9729 |
| D richness | Spring | Flow regime | 1 | 7 | 0.03 | 0.8636 |
|  | Autumn | Flow regime | 1 | 8 | 0.69 | 0.4309 |
| Total abundance | Spring | Flow regime | 1 | 7 | 1.48 | 0.2626 |
|  | Autumn | Flow regime | 1 | 8 | 0.31 | 0.5955 |
| EPT abundance | Spring | Flow regime | 1 | 7 | 0.81 | 0.3972 |
|  | Autumn | Flow regime | 1 | 8 | 6.03 | ***0.0396*** |
| OCH abundance | Spring | Flow regime | 1 | 7 | 11.98 | ***0.0105*** |
|  | Autumn | Flow regime | 1 | 8 | 7.88 | ***0.0229*** |
| D abundance | Spring | Flow regime | 1 | 7 | 1.39 | 0.2767 |
|  | Autumn | Flow regime | 1 | 8 | 0.07 | 0.7928 |
| Aquatic passive | Spring | Flow regime | 1 | 7 | 0.07 | 0.7296 |
|  | Autumn | Flow regime | 1 | 8 | 0.02 | 0.8294 |
| Aquatic active | Spring | Flow regime | 1 | 7 | 0.97 | 0.3572 |
|  | Autumn | Flow regime | 1 | 8 | 1.84 | 0.2122 |
| Aerial passive | Spring | Flow regime | 1 | 7 | 4.41 | 0.0740 |
|  | Autumn | Flow regime | 1 | 8 | 0.03 | 0.8648 |
| Aerial active | Spring | Flow regime | 1 | 7 | 16.80 | ***0.0046*** |
|  | Autumn | Flow regime | 1 | 8 | 5.66 | ***0.0446*** |
| Eggs and statoblasts | Spring | Flow regime | 1 | 7 | 1.09 | 0.3309 |
|  | Autumn | Flow regime | 1 | 8 | 3.43 | 0.1013 |
| Cocoons | Spring | Flow regime | 1 | 7 | 0.49 | 0.5060 |
|  | Autumn | Flow regime | 1 | 8 | 0.24 | 0.6373 |
| Diapause or dormancy | Spring | Flow regime | 1 | 7 | 39.39 | ***0.0004*** |
|  | Autumn | Flow regime | 1 | 8 | 2.29 | 0.1653 |
| No resistance form | Spring | Flow regime | 1 | 7 | 12.62 | ***0.0093*** |
|  | Autumn | Flow regime | 1 | 8 | 0.54 | 0.4834 |
