# Supplementary material for: Understanding the effects of predictability, duration, and spatial pattern of drying on benthic invertebrate assemblages in two contrasting intermittent streams
Source: PLoS One. 2018 Mar 28;13(3):e0193933. doi: 10.1371/journal.pone.0193933 (PMC5874014; doi:10.1371/journal.pone.0193933)
Supplement: S7 Table — (EPT: Ephemeroptera, Plecoptera and Trichoptera, OCH: Odonata, Coleoptera and Heteroptera). (DOCX) [file pone.0193933.s009.docx]

**S7 Table**.

|  |  |  | **Solutions for Fixed Effects** | | | | | |
| --- | --- | --- | --- | --- | --- | --- | --- | --- |
| **Stream** | **Season** | **Metric** | **Effect** | **Estimate** | **Standard Error** | **DF** | **t Value** | **Pr > \|t\|** |
| Rogativa | Spring | **EPT abundance (log)** | **Intercept** | 67.932 | 0.8391 | 6.94 | 8.1 | <0.0001 |
|  |  |  | **Distance (km)** | -13.049 | 0.3668 | 7.074 | -3.56 | 0.0091 |
|  |  | **OCH abundance (log)** | **Intercept** | 1.851 | 0.6562 | 5.743 | 2.82 | 0.0318 |
|  |  |  | **Dry (%)** | 38.621 | 13.507 | 5.674 | 2.86 | 0.0307 |
|  |  |  | **Distance (km)** | -0.7614 | 0.2792 | 5.795 | -2.73 | 0.0356 |
|  |  | **Aerial active** | **Intercept** | 455.189 | 27.655 | 7.1 | 16.46 | <0.0001 |
|  |  |  | **Distance (km)** | -34.503 | 12.166 | 7.328 | -2.84 | 0.024 |
