# Supplementary material for: Understanding the effects of predictability, duration, and spatial pattern of drying on benthic invertebrate assemblages in two contrasting intermittent streams
Source: PLoS One. 2018 Mar 28;13(3):e0193933. doi: 10.1371/journal.pone.0193933 (PMC5874014; doi:10.1371/journal.pone.0193933)
Supplement: S8 Table — (EPT: Ephemeroptera, Plecoptera and Trichopetera, OCH: Odonata, Coleoptera and Heteroptera). (DOCX) [file pone.0193933.s010.docx]

**S8 Table.**

| **Stream** | **Season** | **Variable** | **Equation** |
| --- | --- | --- | --- |
| Rogativa | Spring | EPT abundance (log) | 6.793 – 1.305 Dis |
|  | Spring | OCH abundance (log) | 1.851 + 3.862 Dry -0.761 Dis |
|  | Spring | Aerial active | 45.519 – 3.450 Dis |
